# Is Abdominal Cocoon a Sequela in Recovered Cases of Severe COVID-19?

**DOI:** 10.7759/cureus.22384

**Published:** 2022-02-19

**Authors:** Juwairiah Abdur Raheem, Suresh C Annu, Lahari Ravula, Sara Samreen, Ariyan Khan

**Affiliations:** 1 Department of General Surgery, Deccan College of Medical Sciences, Hyderabad, IND; 2 Department of Surgical Gastroenterology, Deccan College of Medical Sciences, Hyderabad, IND; 3 Department of General Surgery, Bhaskar Medical College, Hyderabad, IND

**Keywords:** post-covid sequelae, sclerosing encapsulating peritonitis, covid-19 sequela, subacute small bowel obstruction, small-bowel obstruction, severe covid-19, abdominal cocoon

## Abstract

Abdominal cocoon is one of the rare causes of intestinal obstruction mostly diagnosed at the operating table. Its etiology is primarily unknown but can be secondary to known causes. The involvement of the gastrointestinal (GI) system was a common feature during the second wave of COVID-19, and at present, there are reports of GI symptoms in patients who have completely recovered from COVID-19. Abdominal cocoon formation has been reported during the active stage of COVID-19 but not as its sequela. We report two cases with a high degree of suspicion of abdominal cocoon formation in middle-aged individuals with no comorbidities, who recovered from a severe form of COVID-19.

## Introduction

Coronavirus disease (COVID-19) is a respiratory disease of infectious origin, caused by the virus SARS-CoV-2. Though it primarily affects the respiratory system, it has been proven to involve other systems of the human body such as the gastrointestinal (GI), cardiovascular and nervous systems [1]. The incidence of GI symptoms after complete recovery from COVID-19 is reported to be 44% [2]. An abdominal cocoon is a rare cause of bowel obstruction, wherein a cluster of bowel loops are enclosed within a thick fibro-collagenous membrane. It often presents with certain specific and non-specific symptoms [3]. The specific manifestations are progressive intermittent colicky abdominal pain with gradually increasing intolerance to oral intake of solids, followed by semi-solids and liquids. The non-specific symptoms include weight loss, anorexia, nausea, palpable abdominal mass, and vague abdominal pain [4]. Most patients are asymptomatic or present with non-specific symptoms, leading to misdiagnosis and ill-treatment. We report two cases of an abdominal cocoon in recovered patients of severe COVID-19 with no comorbidities and propose a high degree of suspicion between COVID-19 and the formation of the abdominal cocoon.

## Case presentation

Case 1

**Figure 1:**
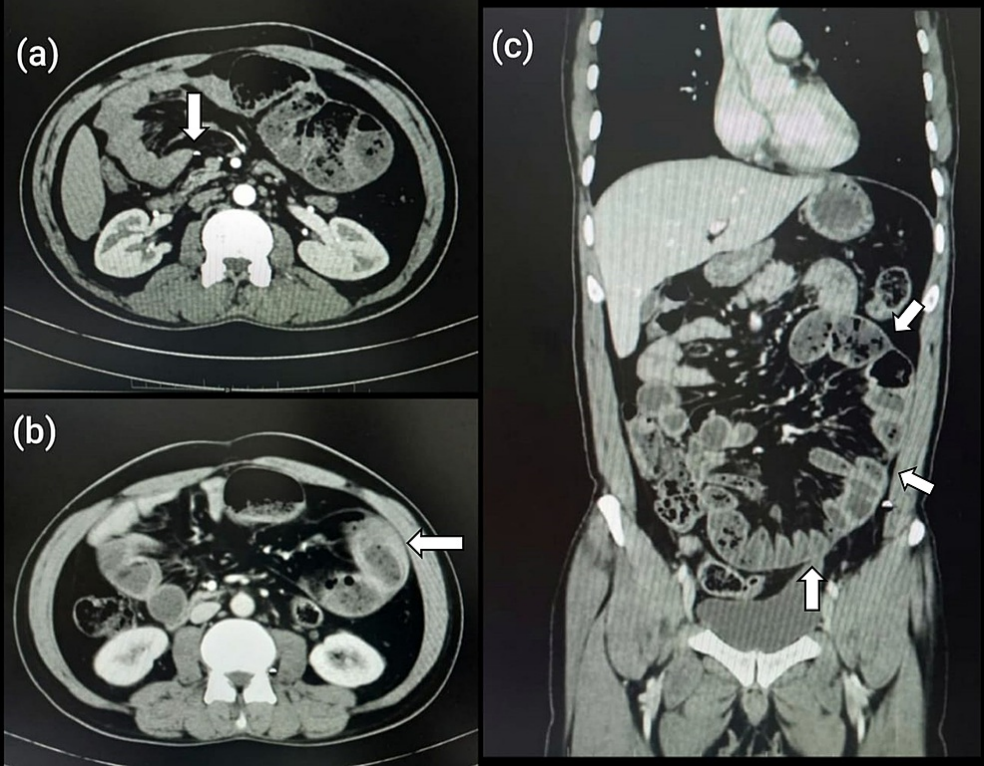
CECT abdomen images (a) Axial section with the arrow showing obstruction in the jejunum. (b) Axial section with the arrow showing venous congestion around dilated loops. (c) Coronal section with arrows showing cocoon outline with dilated bowel loops. CECT - Contrast-enhanced computed tomography

A 46-year-old gentleman with nil comorbidities presented to the outpatient department with complaints of colicky abdominal pain, for the last four months which was managed conservatively on multiple occasions. He complained of nausea and one episode of vomiting two days back along with constipation. The patient had an attack of COVID-19 (CORADS-5) six months back for which he required Intensive Care Unit (ICU) admission with ventilatory support for three weeks. There is no past or contact history of pulmonary tuberculosis and no history of previous surgeries. Vitals were normal and abdominal examination revealed tenderness in the umbilical region. Biochemical and hematological parameters were within normal limits. Contrast-enhanced computed tomography (CECT) revealed small bowel obstruction with signs of venous congestion possibly due to trans-mesenteric small bowel internal hernia (Figures 1a-1c). 

**Figure 2:**
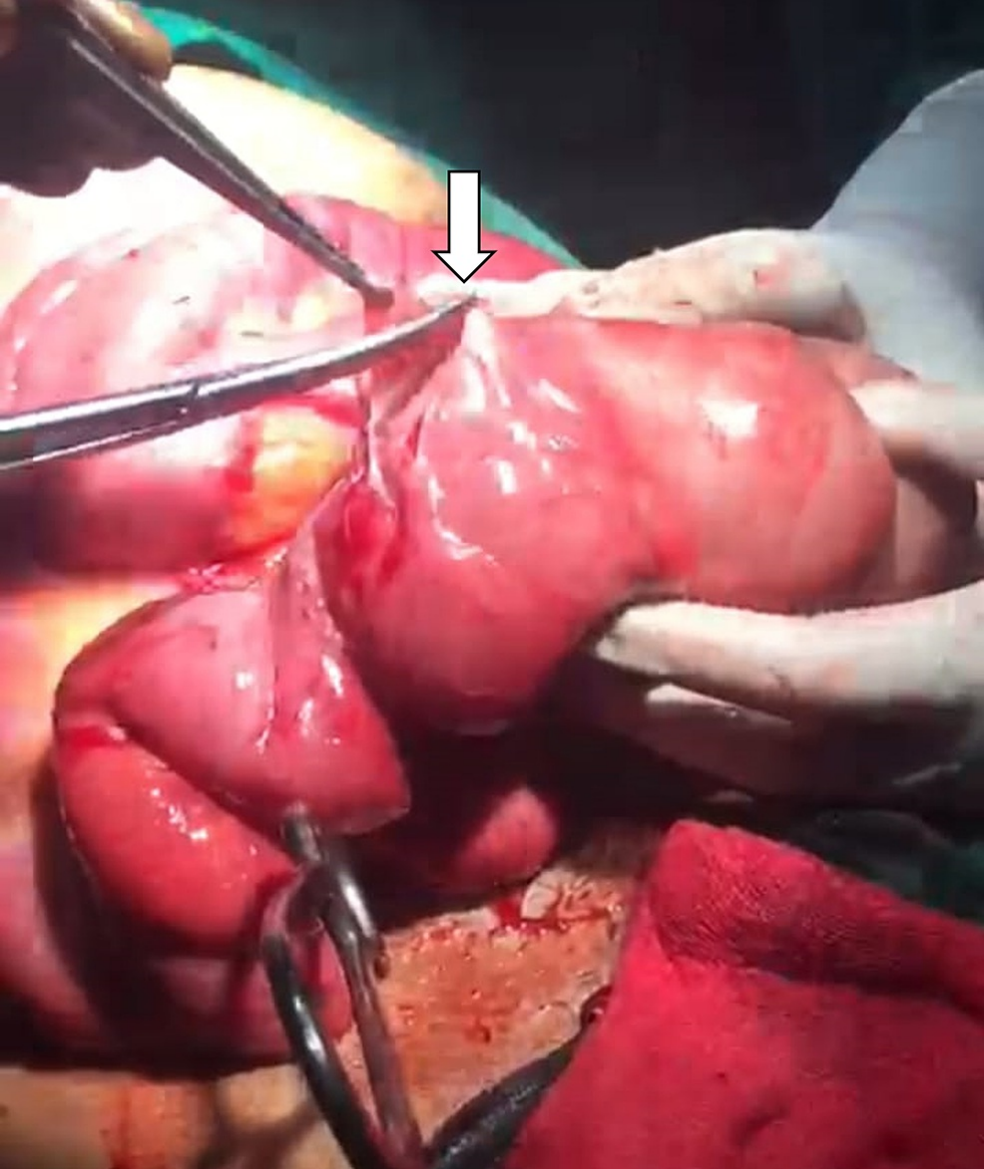
Excision of cocoon sac; arrow showing the opening of sac

The patient was planned for exploratory laparotomy. Intraoperative findings revealed cocoon formation in the right iliac fossa and right lumbar with grade 1 and 2 adhesions (Figure 2). 

The contents were ileal and jejunal loops along with mesentery. The remaining viscera was normal. The post-operative period was uneventful with dramatic clinical improvement and the patient was discharged on postoperative day 6.

Case 2

**Figure 3:**
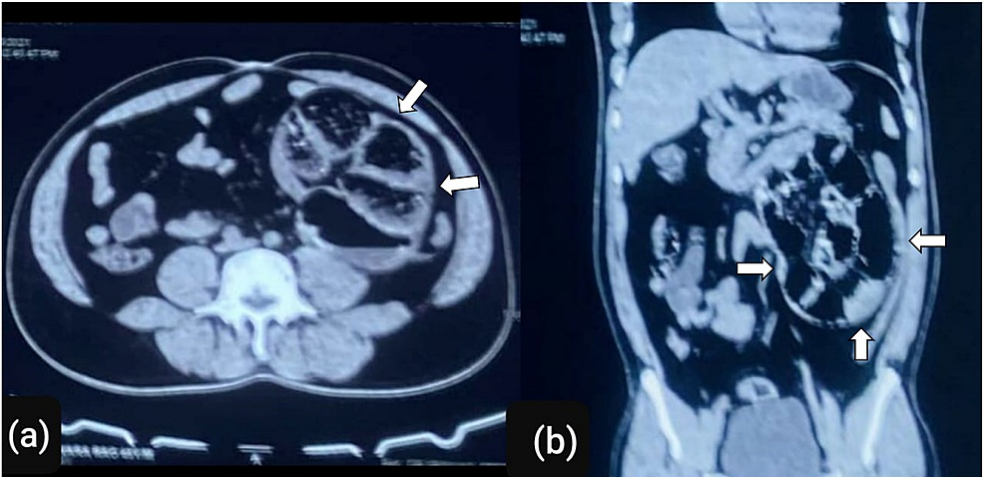
(a) Axial and (b) coronal sections of the abdomen showing internal hernia with closed-loop small bowel obstruction on the left side

A 47-year-old gentleman with nil comorbidities presented with the complaints of left upper colicky abdominal pain, nausea, vomiting, and loss of appetite for one week. The pain was acute in onset, severe, and associated with two episodes of vomiting. The patient noticed a non-tender, lump in the left upper quadrant. The patient was diagnosed with COVID-19 (CORADS-5) six months back, for which he required ICU admission and ventilatory support for three weeks. No history of pulmonary tuberculosis, peritoneal dialysis, or surgeries. There is a history of similar episodes three months back which was managed conservatively. Abdominal examination revealed a distended abdomen with a palpable, non-tender intraperitoneal mass about 5*6cm in the left lumbar region with absent bowel sounds. CT abdomen and pelvis with and without contrast revealed dilated small bowel loops (jejunum) showing fluid-fluid level and small bowel feces sign forming a closed sac-like structure in the left abdominal cavity (Figures 3a, 3b).

**Figure 4:**
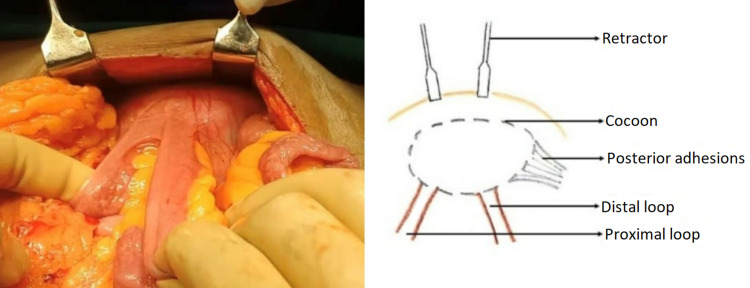
Schematic intraoperative representation of abdominal cocoon with proximal and distal loops

Emergency exploratory laparotomy was done and intraoperatively an opaque membrane encasing part of the small bowel suggestive of the abdominal cocoon with dense interloop adhesions which were also extending posteriorly (Figure 4). 

The part encased had viable jejunal loops with no significant change in the caliber of proximal and distal loops (Video 1).

**Video 1:** Intraoperative abdominal cocoon with proximal and distal loops

Hence only adhesiolysis was done and the sac excised was sent for histopathological examination (HPE). HPE showed congested fibroadipose tissue with hemorrhage and mild inflammation, negative for granuloma, atypia or malignancy. The postoperative stay was uneventful and the patient was discharged on postoperative day 7.

## Discussion

We report two cases of abdominal cocoon occurring as a sequela in recovered severe COVID-19, diagnosed in middle-aged individuals with no comorbidities or any known cause of abdominal cocoon and negative RT-PCR on admission. There have been reports of abdominal cocoon formation during the active stage of COVID-19 [5,6], but none as a sequela of COVID-19, and this is the first case report to the best of our knowledge. In a report by Mofti et al. [5], the patient recovered from severe COVID-19, was about to be discharged when he experienced abdominal discomfort associated with nausea and decreased bowel movements, whereas in our case the patient presented with symptoms six months after recovery from severe COVID-19.

It is well known that coronavirus primarily attacks the respiratory system but also affects various other systems of the body. The prevalence of GI symptoms during the active stage of COVID-19 is recorded to be more than 50%, of which loss of appetite and anorexia are most common [7]. Patients who suffered from severe COVID-19 are at high risk for developing unusual GI complications such as ileus, hepatic necrosis, acalculous cholecystitis, bowel ischemia, and GI bleeding [8,9]. A wide variety of COVID-19 sequelae involving various systems have been reported to date. Reported GI COVID-19 sequelae are spleen abscess and liver abscess with necrosis [10,11]. An abdominal cocoon is a rare cause of intestinal obstruction, where the small bowel is enclosed by a fibro-collagenous membrane [3]. Apart from the small bowel, it can also contain the large intestine, liver, or stomach as its contents [12]. Risk factors that are associated with potential long-term COVID-19 sequela include the severity of disease (SpO_2_<94% and patient requiring ICU support and mechanical ventilation) and the presence of comorbidities particularly hypothyroidism [13,14]. Around 44% of individuals, who had successfully recovered from COVID-19, presented with fresh GI symptoms almost three months after discharge [2]. The most common GI symptoms noted after recovery from COVID-19 are loss of appetite, nausea, acid reflux, diarrhea, and the less common GI symptoms include abdominal distension, belching, vomiting, abdominal pain, and bloody stools [2], few of which were also noted in our study. There is an occurrence of the multisystem inflammatory syndrome and autoimmune conditions after COVID-19 infection as reported by the Centers for Disease Control and Prevention [15]. The correlation between COVID-19 and abdominal cocoons was suspected due to similar case reports during the peak phase of the pandemic. The exact pathogenesis of abdominal cocoon formation is not known but it is said, that irritation of the peritoneum causes an inflammatory release of cytokines and fibrosis leading to the formation of a dense sac, enclosing clustered bowel loops [16]. The pathogenesis can also be explained on HPE, which shows infiltration with chronic inflammatory cells and proliferation of fibrous and connective tissues [17].

**Figure 5:**
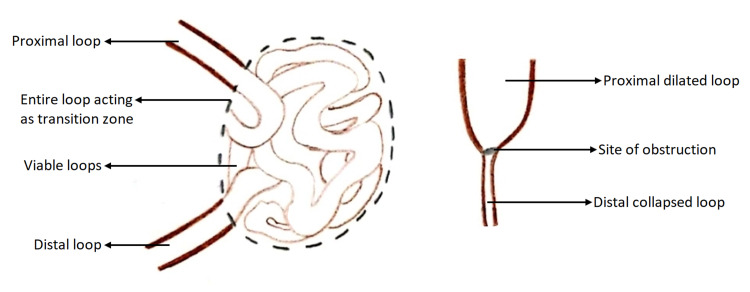
Depicting intestinal obstruction pathology in cocoon and non-cocoon

The pre-operative diagnosis of an abdominal cocoon is a challenge and the majority of these cases are operated on as intestinal obstruction. In our discussion, both cases were of healthy individuals who presented with chronic vague GI symptoms, with a past history of severe COVID-19. The abdomen examination revealed a non-tender lump in only one patient with unremarkable laboratory investigations in both individuals. Few authors have described the CT scan findings of the abdominal cocoons as clustered bowel loops covered by a thin membrane, but by large pre-operative diagnosis by imaging is still difficult [18,19], and most cases are diagnosed on the operating table. The impression given by radiologists herein were small bowel internal hernia and obstruction with the exact cause for obstruction being elusive. Intraoperatively, contrary to routine obstruction, there was no gross proximal dilatation or distal collapse of the bowel loops (Figure 5).

Approximately 1.5 to 2 meters of viable small bowel was found entangled within the sac with no significant change in caliber of the afferent and efferent loops. This could be the reason for its sub-acute presentation and atypical radiological findings.

## Conclusions

Abdominal cocoon is a rare cause of intestinal obstruction and can also be seen in recovered cases of severe COVID-19. The exact mechanism of its formation is unknown, but there are studies suggestive of its formation in the active stage of COVID-19. In our case report, the two patients had a history of severe COVID-19 with no comorbidities and no other history favorable for the formation of an abdominal cocoon. Based on the substantial clinical and surgical correlation, we conclude that abdominal cocoon could be a long-term sequela of recovered severe COVID-19.

This to the best of current evidence is the first case to document abdominal cocoon as a sequela in recovered severe COVID-19 patients and thus, requires further research work and documentation.

## References

[1] Behzad S, Aghaghazvini L, Radmard AR, Gholamrezanezhad A (2020). Extrapulmonary manifestations of COVID-19: radiologic and clinical overview. Clin Imaging.

[2] Weng J, Li Y, Li J (2021). Gastrointestinal sequelae 90 days after discharge for COVID-19. Lancet Gastroenterol Hepatol.

[3] Singhal M, Krishna S, Lal A (2019). Encapsulating peritoneal sclerosis: the abdominal cocoon. Radiographics.

[4] Singh B, Gupta S (2013). Abdominal cocoon: a case series. Int J Surg.

[5] Mofti AH, Ghabashi FA, Sadagah MM (2021). Sclerosing encapsulating peritonitis following recovery from COVID-19 pneumonia. Cureus.

[6] Yusuf MH (2021). Sclerosing encapsulating peritonitis: a rare cause of intestinal obstruction. Cureus.

[7] El Ouali S, Achkar JP, Lashner B, Regueiro M (2021). Gastrointestinal manifestations of COVID-19 [PREPRINT]. Cleve Clin J Med.

[8] Patel S, Parikh C, Verma D (2021). Bowel ischemia in COVID-19: a systematic review. Int J Clin Pract.

[9] Martin TA, Wan DW, Hajifathalian K (2020). Gastrointestinal bleeding in patients with coronavirus disease 2019: a matched case-control study. Am J Gastroenterol.

[10] AlZarooni N, AlBaroudi A, AlOzaibi L, AlZoabi O (2021). Splenic abscess as a possible sequela of COVID-19: a case series. Ann Saudi Med.

[11] Liemarto AK, Budiono BP, Chionardes MA, Oliviera I, Rahmasiwi A (2021). Liver abscess with necrosis in post COVID-19: a case report. Ann Med Surg (Lond).

[12] Tannoury JN, Abboud BN (2012). Idiopathic sclerosing encapsulating peritonitis: abdominal cocoon. World J Gastroenterol.

[13] Kamal M, Abo Omirah M, Hussein A, Saeed H (2021). Assessment and characterisation of post-COVID-19 manifestations. Int J Clin Pract.

[14] Naik S, Haldar SN, Soneja M (2021). Post COVID-19 sequelae: a prospective observational study from Northern India. Drug Discov Ther.

[15] (2022). Centers for Disease Control and Prevention: Post-COVID Conditions: Information for Healthcare Providers. https://www.cdc.gov/coronavirus/2019-ncov/hcp/clinical-care/post-covid-conditions.html.

[16] Oran E, Seyit H, Besleyici C, Ünsal A, Alış H (2015). Encapsulating peritoneal sclerosis as a late complication of peritoneal dialysis. Ann Med Surg (Lond).

[17] Arif SH, Mohammed AA (2019). Abdomen cocoon causing chronic abdominal pain and intestinal obstruction; a case series. Ann Med Surg (Lond).

[18] Hamaloglu E, Altun H, Ozdemir A, Ozenc A (2002). The abdominal cocoon: a case report. Dig Surg.

[19] Hur J, Kim KW, Park MS, Yu JS (2004). Abdominal cocoon: preoperative diagnostic clues from radiologic imaging with pathologic correlation. AJR Am J Roentgenol.

